# Energy Metabolism Decline in the Aging Brain—Pathogenesis of Neurodegenerative Disorders

**DOI:** 10.3390/metabo10110450

**Published:** 2020-11-07

**Authors:** Janusz Wiesław Błaszczyk

**Affiliations:** Department of Human Motor Behavior, Jerzy Kukuczka Academy of Physical Education, 40-065 Katowice, Poland; j.blaszczyk@awf.katowice.pl

**Keywords:** brain aging, energy metabolism, neurodegeneration, neurodegenerative disorders

## Abstract

There is a growing body of evidencethat indicates that the aging of the brain results from the decline of energy metabolism. In particular, the neuronal metabolism of glucose declines steadily, resulting in a growing deficit of adenosine triphosphate (ATP) production—which, in turn, limits glucose access. This vicious circle of energy metabolism at the cellular level is evoked by a rising deficiency of nicotinamide adenine dinucleotide (NAD) in the mitochondrial salvage pathway and subsequent impairment of the Krebs cycle. A decreasing NAD level also impoverishes the activity of NAD-dependent enzymes that augments genetic errors and initiate processes of neuronal degeneration and death.This sequence of events is characteristic of several brain structures in which neurons have the highest energy metabolism. Neurons of the cerebral cortex and basal ganglia with long unmyelinated axons and these with numerous synaptic junctions are particularly prone to senescence and neurodegeneration. Unfortunately, functional deficits of neurodegeneration are initially well-compensated, therefore, clinical symptoms are recognized too late when the damages to the brain structures are already irreversible. Therefore, future treatment strategies in neurodegenerative disorders should focus on energy metabolism and compensation age-related NAD deficit in neurons. This review summarizes the complex interrelationships between metabolic processes on the systemic and cellular levels and provides directions on how to reduce the risk of neurodegeneration and protect the elderly against neurodegenerative diseases.

## 1. Energy Metabolism of the Brain

Life is a complex form of energy exchange, and energy metabolism is considered the foundation of life [1]. Its major role is to maintain life-sustaining metabolic processes. The human brain is the prime controller of organismal metabolism, including its tissue. It consumes approximately 20% of organismal energy, although its mass comprises approximately 2% of the body’s mass [2,3,4,5]. The energy is used mainly for the generation and transmission of action potentials, as well as the release of neurotransmitters at synaptic junctions [6,7,8,9,10]. The energy at the cellular level is necessary for setting resting gradients of ion concentration. In particular, the neuronal and mitochondrial resting potentials are determined by specific gradients of sodium, potassium, and chloride ions.The gradients strictly depend on the ATP-controlled ion pumps and transporters.

Brain metabolism requires the delivery of nutrients and energy fuel from outside [4,11,12,13,14,15]. Each limitation of essential nutrients, or decline of their absorption, results in a deficiency state that compromises cellular growth, function, and survival [11,12]. Generally, cellular energy metabolism depends on several factors, such as the supply of substrates and the efficiency of their transport to the cytoplasm and mitochondria [15,16,17,18,19,20,21]. Additionally, the kinetics of all cyclic intracellular reactions depends on tissue temperature and pH of the cytoplasm [22]. The physicochemical factors determine the efficiency of metabolic reactions. At the same time, some energy reserves must be allocated for the removal of soluble waste proteins and metabolic products [23,24].

Brain energy metabolism relies on two main substrates: glucose and oxygen [4,11,12,13]. Neurons require, on average, six molecules of oxygen per molecule of glucose [11,12,13], whereas the number of oxygen molecules in the arterial blood exceeds the number of glucose molecules by only a factor of 1.5 [11]. To solve this problem, the neuronal intake of glucose is limited by ATP production—which, in turn, depends on oxygen delivery. Both oxygen and glucose must be delivered to the brain continuously by the cerebral blood flow (CBF) [11,12,13,25,26]. The inflow of glucose to the brain depends on the concentration gradient between blood and brain tissue [27]. Therefore, even a large increase in the cerebral blood flow does not substantially affect the brain’s glucose level [13,28]. In contrast, CBF regulates oxygen availability for the brain [11,13]. During energy production, oxygen is almost fully reduced to water, and only 1–2% of the O_2_ is reduced incompletely to give the superoxide anions [18,19,20,21,29]. Increasing with age, excessive production of free radicals impairs mitochondrial functioning by causing oxidative damage to macromolecules [18,19,20,21,29], and eventually contributing to neuronal death [30,31,32].

Mitochondrial processes convert fuel energy of oxygen and glucose into chemical, thermal, and electric energy, as well as water [32,33]. Part of the energy is allocated to waste product removal [23,24]. Additionally, in the aging brain, an increasing amount of energy is allocated to repairerroneous or abnormal metabolic processes. Therefore, we can assess physiological and pathological brain status based on the overall energy balance [21,33].

The energy production and storage in the electric field are specific for neuronal mitochondria [19,34,35]. These semi-autonomous organelles bounded in double-membrane are found particularly abundantly in axons near the Ranvier nodes and synaptic junctions [34,36]. The distribution of mitochondria can vary substantially in number, size, and membrane potential depending on differences in recirculated ATP levels, and thus, energetic processes [4,7,34]. Mitochondrial dysfunctions result in a decline in ATP production, which, in turn, reduces glycolysis, increases oxidative stress, and limits the self-repair capability of neurons—leading to excessive neuronal apoptosis culminating in neurodegenerative disorders [18,32,35,37]. Particularly, the accumulation of mitochondrial DNA mutations accelerates normal aging, leads to oxidative damage to nuclear DNA, and impairs gene transcription [19,29]. In consequence, it requires an intensification of the NAD-dependent repair enzymes, which additionally impoverishes the intraneuronal NAD pool [38,39,40,41,42,43,44].

**Figure 1:**
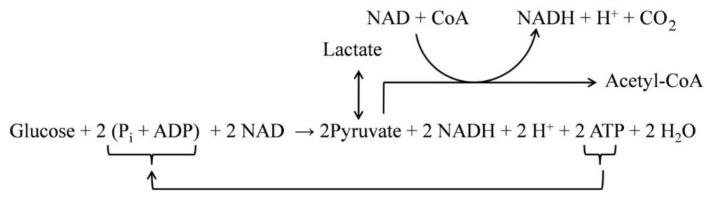
Simplified diagram presenting participation of the ATP in glycolysis—the input pathway in energy metabolism in neurons (for review see Reference [45]). The ATP produced in the mitochondrial process of oxidative phosphorylation is used in neurons as the rate-limiting factor of glycolysis. In this entry process, each molecule of glucose 6-phosphate is broken down into two molecules of pyruvate, which are then used as a source of energy.The chronic decline in ATP production results in neuronal insensitivity to glucose that eventually terminates the cellular energy metabolism.

Glucose supplies energy to neurons through the glycolytic pathway that converts glucose into pyruvate, and hydrogen ions [27,45,46] (see Figure 1). Glycolysis is one of the two main metabolic pathways in neuronal energy metabolism. As glucose enters neurons, it is phosphorylated by ATP to glucose 6-phosphate (G6P). It is a necessary and irreversible first step of neuronal energy metabolism, which is controlled by ATP feedback. The level of the ATP-derived phosphoric groups is the main regulator of glucose flux to glycolysis. In this entry process, each molecule of glucose 6-phosphate is broken down into two molecules of pyruvate, which are then used as a source of energy [47].

The process of glucose phosphorylation determines its fate in cellular metabolism. Glucose itself may easily diffuse across the cellular membrane [28,46,48,49,50], but its phosphorylated form (G6P), however, cannot exit the cytoplasm and must enter the metabolic pathway of glycolysis. Due to ATP-feedback control of glucose input, even in the case of an increased level of blood glucose, e.g., after a meal, only limited by the ATP amount of glucose can be used by the brain for energy metabolism [12,25,27,46]. The rest of it is stored in the form of glycogen, mostly in skeletal muscles, liver, and adipose tissue.

A greater level of G6P, in addition to the free inflow of oxygen, adjusts ATP production proportionally to neuronal activity, increasing the metabolism of glucose [51]. This also sets a level of neuronal oxygen needs. Both glucose and oxygen metabolic pathways closely interact, forming, what is called, the ATP-controlled glucose-oxygen metabolic synergy. Other substrates, such as lactate, may support neuronal energy metabolism to a limited extend [52,53]. In increased concentrations of lactate, the enzyme exhibits feedback inhibition and lowers the rate of the pyruvate to lactate conversion.

Pyruvate supplies energy to neurons through the Krebs cycle only when oxygen is present [45]. It is converted into acetyl-coenzyme A, which is the main input for the Krebs cycle in mitochondria. The main synthesis of ATP is initiated by the oxidation of nicotinamide adenine dinucleotide (NADH) and the reduction of O_2_ in the electron transport chain [14]. NAD is the main coenzyme in redox reactions in mitochondria [54,55,56,57,58]. During the reduction of NAD, the molecule acquires two electrons and one proton, while the second proton is released into the cytoplasm. Under physiological conditions, neurons can recover most of the used NAD in the salvage pathway, and only limited amounts of NAD is delivered by the *de novo* pathway [57]. The human brain depends primarily on nicotinamide riboside (NR) and tryptophan as precursors for NAD de novo synthesis [59]. In a normal healthy brain, the level of NAD exceeds its neuronal needs [58]. The level, however, declines with age and particularly is reduced in various chronic diseases [42,56,58].

The electron transport chain (ETC) is a series of complexes that control the mitochondrial transfer of electrons from donors to acceptors via redox reactions. The electrons are carried from NADH donors, through a chain of electron carriers, to the final acceptor, the oxygen. During this process, two gradients are built upon the inner mitochondrial membrane. First is the oxygen-dependent electron gradient that results in the negative polarization of the mitochondrial matrix relative to the neuronal cytoplasm. The negative polarization of the matrix attracts positively charged protons (H^+^) toward the outer surface of the inner mitochondrial membrane. Both electron and proton gradients produce a strong electric field that presses protons into the inner mitochondrial membrane [60]. The magnitude of the resultant electric field may eventually force the protons to break the inner mitochondrial membrane, thus making electropores, i.e., the functional proton channels. While passing the inner mitochondrial membrane, the proton current is driving a “molecular pump,” using the enzyme ATP synthase, to produce an ATP. It converts the energy of the protons to the chemical energy of ATP. Theoretically, at least three protons must pass the inner mitochondrial membrane to produce one ATP molecule. The process of electroporation allows protons entering the mitochondrial matrix and reacts with the oxygen. The end products of this process are water and heat. In the case of unbalanced electron and proton currents, some oxygen molecules remain unused and are precursors of reactive oxygen species (ROS) [21]. During energy production, oxygen is almost fully reduced to water, and only 1–2% of the O_2_ is reduced incompletely to give the superoxide anions [18]. Increasing with age, excessive production of ROSimpairs mitochondrial functioning by causing oxidative damage to macromolecules [18,19,20,21,29], and eventually contributing to neuronal death [21,29,30,31,32,33,38].

Metabolic homeostasis of the brain is heavily regulated and involves close interaction of neurons and glial cells, which together form functional metabolic units [6,10,26]. The existence of the ATP-controlled glucose-oxygen synergy in neurons and glial cells is well documented experimentally [11,12,13,25,50,51,53,61]. The activity of enzymes and proteins associated with glucose supply and glycolysis is adjusted to the availability of oxygen [13]. A low level of oxygen activates hypoxia-inducible factor 1 (HIF-1) that, in turn, upregulates the glucose transporters GLUT 1 and GLUT3, which are responsible for basal glucose uptake and activity of glucose 6-phosphate isomerase [13,50,53,62,63]. Both transporters intensify anaerobic glycolysis and help overcome the hypoxia crisis. Similarly, hypoglycemia augments cellular glucose transport and metabolism, with a specific increase in the activity of glucose transporters GLUT-1 andGLUT-3 [53]. GLUT-1 transporters are located in the endothelial cells lining the brain microvasculature, glial cells, and choroid plexus, whileGLUT-3 is expressed in neurons [48,49,50,53,63]. Both isoforms meet the energy demands of the brain by transporting glucose into the central nervous system in an insulin-independent manner [53,64]. GLUT-3 activity is critical in protecting against hypoglycemia [28,49,63]. A transient increase in the activity of GLUT-3, after either hypoxic ischemia or hypoxia, attempts to preserve the cellular glucose supply, thereby protecting against the depletion of cellular ATP stores [53]. Therefore, an increase in GLUT-3 is the brain-protective mechanism that may inhibit neuronal death [21,65].

Generally, dysfunction of brain energy metabolism may result from a decline of neural or central metabolic controls. The latter is responsible for the organismal aging process. Functional deficits, due to metabolic dysfunction at the cellular level, are very common and can be efficiently compensated by plastic changes, including synaptogenesis and partially by local neurogenesis. In the next parts of the review, I will summarize our knowledge, referring to central and local mechanisms of brain aging.

## 2. The Self-Control of the Brain’s Metabolism, Life, and Aging

Aging and death are natural processes necessary for species adaptation, and development. Their course is driven by individually shaped energy metabolism [66,67]. The energy metabolism undergoes significant changes in the course of ontogeny. A growing body of evidence suggests that the aging process is initiated and controlled centrally by the age-related decline of the hypothalamus functioning [68,69,70]. Consequently, the energy metabolism on organismal, tissue, and cellular levels is gradually extinguished, culminating in apoptosis-the programmed cell death [30,31,65,68].

Understanding mechanisms of the energy metabolism control at the hypothalamus level are, thus, the first step for controlling them that can open new perspectives in the prevention of neurodegenerative diseases [68,71]. The hypothalamus controls most of the metabolic processes and many functions of the autonomic and the central nervous system that are fundamental for life-sustaining [51,72,73,74,75,76]. It produces and secretes neurohormones, and thus, functional connects through the pituitary gland the central nervous system with the endocrine system. In this way, the hypothalamus maintains energy balance by controlling hunger, thirst, circadian rhythm, sleep, and body temperature [27,73,74,75]. The lateral hypothalamus, also known as the lateral hypothalamic area (LHA), is the orexinergic nucleus that has extensive projections throughout the nervous system [75]. This system mediates several cognitive and motor processes, such as agitation, feeding behavior, digestive functions, pain sensation, control of body temperature, blood pressure, and many others [75,76]. Clinically relevant disorders involving the dysfunction of the orexinergic projection system include narcolepsy, motility disorders, or functional gastrointestinal disorders, including visceral hypersensitivity and eating disorders. Interestingly, sleep disorders, one of the prodromal symptoms of Parkinson’s disease [72,77,78,79], are associated with a marked reduction in the population of LHA orexinergic projection neurons and lowered the level of orexin peptides in the cerebrospinal fluid [3,9,76,77,78,79,80].

Hypothalamic neurons integrate systemic energy homeostasis and neuroendocrine functions [75,80,81,82]. The orexigenic neurons are involved in food intake motivation and reward value [80,81,82]. The hypothalamic-pituitary-adrenal axis regulates stress levels, and the hypothalamic-pituitary-thyroid axis is responsible for metabolism control and regulating visceral functions [74,80]. Histaminergic, dopaminergic, serotoninergic, noradrenergic, and cholinergic nuclei, to which the lateral hypothalamus orexin neurons project, form the activating network of the reticular formation located throughout the brainstem, and which determines the subjective quality of life. Moreover, the projection of the lateral hypothalamus to the ventral tegmental area (VTA) controls the oxytocin reward system establishing positive social relationships, such as feelings of friendship, love, and sympathy. Glutamate, endocannabinoids, and neuropeptides (orexin-A and orexin-B), are here the primary signaling neurotransmitters. The cannabinoid receptor 1 (CB1) is co-localized in many output structures of the LHA orexinergic projection, which can explain the universal therapeutic properties of marijuana, its psychoactive effect, and high efficiency in suppressing convulsive seizures caused by hypoglycemia or insulin resistance.

Susceptibility to neurodegenerative changes is largely dependent on the number of neurons making up a given brain structure. From this perspective, it is obvious that the smaller the number of neurons, the more susceptible to involution of its structure. Unfortunately, there are only 10,000–20,000 orexinergic neurons in the human brain [75]. Their population is reduced by nearly 50% in the process of aging, which may explain why patients with Alzheimer’s disease have reduced levels of orexin in the cerebrospinal fluid [83,84]. Simultaneously, there is observed neurodegeneration of the suprachiasmatic nucleus—another small (containing only 20,000 neurons)—hypothalamic center regulating the circadian rhythm. This may explain why patients with neurodegenerative diseases have sleep and circadian rhythm disorders [72,77]. Practically, almost all PD patients develop sleep disturbances at the early stage of the disease [72,76,78]. The disturbances have a multifactorial etiology, but early degeneration of the *hypothalamus* [76,78,82], *locus coeruleus* noradrenergic system [85], and the serotonergic system related to the *dorsalraphe nucleus* [86] may be of particular importance for the pathology. Furthermore, these age-related brain dysfunctions result in a decline of the restorative function of sleep that may cause the accumulation of potentially neurotoxic waste products within the CNS [87,88]. This, in turn, may escalate and also limits neurogenesis in the aging brain. Finally, the depletion of the hypothalamus function in the process of brain aging is associated with mental changes, which are reflected in a decrease in the subjective value of life and the development of depression, common symptoms of neurodegenerative diseases [89,90,91].

The hypothalamic atrophy appears in the early clinical stages, which suggests that the lesions are a significant cause of neurodegenerative changes [86,89,90,91]. Several preclinical and clinical data indicate that declining energy metabolism intensifies the progress of neurodegenerative processes [67,92]. The impoverished hypothalamic functions primarily lead to energy homeostasis disorders [67]. In brain imaging studies, hypothalamic atrophy (more than 10% by volume) was documented in patients with Alzheimer’s disease [83,93,94].

## 3. Life and Aging of Neurons

The regulation and maintenance of cellular metabolism are a critical challenge for the nervous system [6,16,95]. The metabolic cost of performing and maintaining basic neural functions is disproportionately high, primarily due to the highly complex morphology of neurons, their finely regulated transmembrane ion gradients, and the constant activity of billions of synapses [4,6]. Synaptic transmission is a highly energy-consuming process which uses 80% of the energy necessary for the functioning of the neuronal networks [4,7,16,17,96,97,98,99]. At the same time, neurogenesis, synaptogenesis, and synaptic pruning are the main mechanisms of neuronal plasticity. They determine the brain’s capabilities in learning and memory. Their activity relies mostly on ATP-controlled glucose-oxygen synergy, thus in the aging brain, the mechanisms of learning and memory are impaired first [2,4,16,34,36].

A key to understanding this phenomenon is the close interaction between neuronal activity and their trophic/metabolic processes. Generally, greater neurons and active within their optimal ‘physiological timing’ (including the refractory period)have superior metabolic status and are generally kept in better shape than hypoactive or even hyperactive ones [2,10,12]. Too low or excessive neuronal activity may be destructive since both impair cellular energy metabolism [33,37,39,100,101]. Excessive neuronal activity may initiate the pathological process of excitotoxicity, culminating in the death of the overactive cells [102]. This phenomenon has been observed, among others, in glutaminergic neurons. The exposure of neurons to high levels of glutamate is accompanied by an abrupt opening of calcium channels [103,104]. The increased influx of calcium ions into the cytoplasm activates several cellular enzymes (phospholipases, endonucleases, and proteases) that damage the membranes, cytoskeleton, and cell DNA [105,106,107,108]. Excitotoxicity is thought to accompany many pathological conditions, such as strokes, epilepsy, hearing damage through excessive exposure to noise, and neurodegenerative disorders [102]. Interestingly, other conditions that can lead to excessive levels of glutamate are hypoglycemia and dehydration, both related to faulty energy metabolism.

Fundamental for cellular metabolic processes is nicotinamide adenine dinucleotide (NAD) that participates as a cofactor in cellular energy metabolism [40,58,69,109]. It serves as an electron transporter to power oxidative phosphorylation and ATP production. Besides that, theNADis also the substrate for NAD-consuming enzymes, such asADP-ribose transferases and poly(ADP ribose) polymerases (PARPs), cADPribose synthases, sirtuins, and NAD hydrolase SARM1 [38,39,41,42,110,111,112]. The enzymes mediate several intracellular reactions, including DNA repair, chromatin silencing, transcriptional regulation, metabolic switching, and calcium mobilization. Particularly, sirtuins are NAD-dependent histone deacetylases regulating metabolic function, longevity, and aging [109,111]. PARP over-activation has been associated with dopaminergic neurotoxicity and atrophy [112,113,114], as well as disruption of the mitochondrial ultrastructure [114].

Studies on sirtuins, whose enzymatic activity is closely related to NAD biosynthesis, provided extremely interesting data. Sirtuins regulate the metabolic responses of cells and tissues by adapting them to the level of available nutrients [25,38,111,115,116,117,118]. They also participate in response to cellular stress and in repairing cellular structure damage caused by their metabolism disorders. Since the activity of sirtuins depends on NAD, maintaining the physiological level of NAD in cells plays a critical role in their function [54,55,56,58]. NAD cellular level decline caused by brain aging and during pathological conditions impairs the function of sirtuins. A decline in the energy metabolism of neurons is accompanied by a decrease in their resistance to stress, an increase in damage to the cytoskeleton along with progressive impairment in neuronal processes: bioelectric activity and synaptogenesis.

Sirtuins are the main effectors of the cellular response to metabolism changes and cellular stress [38,69,116]. The key function of nuclear sirtuins is to regulate genome homeostasis under stress. The loss of sirtuin function is associated with genomic instability and deterioration of cell viability, as well as the escalation of neurodegenerative processes. In particular, patients with Alzheimer’s disease have reduced expression of sirtuin 1 (SIRT1) [119]. The physiological activity of SIRT1 can reduce the amount of oligomerized beta-amyloid by increased alpha-secretase synthesis. Thus, SIRT1 promotes brain network functioning and survival [38]. The decrease in SIRT1 synthesis in aging neurons of the cerebral cortex and hippocampus impairs learning and memory, and thus, undermines cognitive functions of the brain. Sirtuin 1 also has an important impact on glucose-induced insulin secretion in pancreatic β-cells, which up to a point, maintains normal brain metabolism. Besides, SIRT1 counteracts insulin resistance of cells in peripheral tissues, including adipose tissue, liver, and skeletal muscle [115].

Sirtuin 1 also improves vascular function by affecting many pathways important for endothelial function. SIRT1 inhibits the expression of inflammatory factors, including interleukin-6 (IL-6), monocyte chemotactic protein 1 (MCP-1), intercellular adhesive molecule 1 (ICAM-1), matrix metalloproteinase 14 (MMP14), and vascular cell adhesion molecule 1 (VCAM-1). Moreover, sirtuin 1 improves blood levels of free fatty acids, triglycerides, cholesterol, and glucose. These protective effects of SIRT1 indicate that it acts as an anti-atherosclerotic agent that slows down the aging process of the brain and the whole body. Thus, nicotinamide mononucleotide therapy may improve the function of blood vessels in older people, partly by activating sirtuin 1 [120].

The sirtuins may affect alleviating the symptoms of depression induced by energy metabolism dysfunctions. Under chronic stress, in the dentate gyrus of the hippocampus, the level of sirtuin 1 and 2 expressions rapidly decline, which is accompanied by symptoms of depression. Supplementation of SIRT1 exerts anti-depressant effects since it is a potent inhibitor of monoamine oxidase A (MAO-A) [55,56,57,121].

Some NAD can be made via the de novo pathway, starting from the essential amino acid tryptophan [55,56,57]. The kynurenine pathway accounts for the catabolism of ingested tryptophan and is the starting point for the biosynthesis of serotonin and melatonin [103,104,107,108]. The kynurenine pathway consists of eight enzymatic steps and one non-enzymatic reaction. At the step catalyzed by the nicotinamide mononucleotide adenylyltransferases, the NAD de novo biosynthesis and NAD salvage pathways converge. In the brain, tryptophan is mainly metabolized via the kynurenine pathway [103,107]. A central compound of the pathway is kynurenine, which can be metabolized in two separate ways. One is furnishing kynurenic acid, and the other 3-hydroxykynurenine and quinolinic acid, the precursors of NAD [18,103,104].

Kynurenic acid is one of the endogenous excitatory amino acid receptor blockers with a high affinity positive modulatory binding site at the AMPA receptor—an ionotropic transmembrane receptor for glutamate that mediates fast synaptic transmission in the CNS. Depending on the tissue type, the kynurenine either continues down its pathway toward the tricarboxylic acid cycle or is transformed into kynurenic acid in microglial cells or astrocytes, respectively [103]. In the CNS, kynurenic acid plays a neuroprotective role. Contrary, the quinolinic acid, which is a biosynthetic precursor to NAD, acts as an agonist of NMDA receptors and neurotoxin [104]. A defect in energy metabolism may lead to neuronal depolarization, excessive activation of NMDA receptors accompanied by an increase in intracellular calcium, and apoptosis [19,29]. There are several neurodegenerative disorders whose pathogenesis has been demonstrated to involve multiple imbalances of the kynurenine pathway metabolism [18].

The kynurenine pathway is an additional source of cellular energy as it can degrade approximately 90% of dietary tryptophan into NAD.Changes in brain tryptophan concentration directly alter the rate of serotonin and quinolinic acid synthesis. Quinolinic acid acts as an agonist of the NMDA receptor, which sets the basic cellular metabolism of neurons. In low concentrations, it is fully catabolized to NAD, thus plays a neuroprotective role. In higher doses, however, it may act as a neurotoxin, gliotoxin, proinflammatory mediator, and pro-oxidant molecule [103,104,108]. Especially, high levels of quinolinic acid appear in the brain in response to inflammation. Pathological levels of the quinolinic acid can impair neuronal function and even trigger apoptosis [18,30,31,33,103,104]. Increased levels of quinolinic acid also destabilize the cytoskeleton of astrocytes and blood vessels endothelial cells, which leads to the degradation of the blood-brain barrier. This, in turn, escalates neuroinflammation and further increases the synthesis of quinolinic acid. Such a pathological sequence escalates neurotoxic effects that accompanied neurodegenerative diseases. Chronic mild stress can increase the metabolism of quinolinic acid in the amygdala and striatum and its reduction in the cingulate cortex. The pathological changes can lead to axonal neurodegeneration in the involved brain areas [104].

Inhibition of the kynurenine pathway results in an additional decline ofNAD level, which correlates with a decrease in cell viability, NAD-dependent SIRT1 activity, and CNS function unless alternative precursors for NAD synthesis are made available [18,103,108]. Excessive activation of the kynurenine pathway, however, increases the neurotoxic activity of quinolinic acids [104]. High quinolinic acid concentrations in cerebrospinal fluids have been observed in several neurodegenerative diseases: Alzheimer’s and Parkinson’s disease, multiple sclerosis, depression, epilepsy, and Huntington’s disease (for review [107]). These findings point to the production of quinolinic acid by the kynurenine pathway as a contributing factor to neurodegenerative diseases.

## 4. Aging of the Brain Circulatory Systems

The extremely limited capability of brain tissue for the storage of oxygen and glucose requires the continuous delivery of both energetic substrates by the cerebral blood flow (CBF) [122,123]. The exclusive ATP production via oxidative phosphorylation may suggest that the CBF response serves both glucose and oxygen delivery increase. Glucose itself contains a moderate amount of chemical energy per bond, as confirmed by the relatively small energy output in glycolysis and the Krebs cycle, which converts glucose to CO_2_ and NADH [61]. The oxidative phosphorylation allows for a large release of free energy from oxygen bonds. This suggests that O_2_, rather than glucose, NAD(H), or ATP, is the molecule that provides the most energy to the brain and is crucial for sustaining its life. Recently, it was shown that only up to 20% of the total brain energy may be provided by mitochondrial oxidation of fatty acids [105].

The circulation of blood and cerebrospinal fluid supply the brain with oxygen, glucose, and nutrients necessary for the life and functioning of neurons [124,125]. Additionally, the circulation of cerebrospinal fluid removes unnecessary and toxic waste [24,83,126]. The central nervous system is unique in being the only system lacking lymphatic vessels to assist in removinginterstitial metabolic waste products. Recent work has led to the discovery of the glymphatic system, a glial-dependent perivascular network that subserves a pseudolymphatic function in the brain [23,24,87,124].

Studies on the glymphatic and meningeal lymphatic systems in humans call for a reevaluation of the physiological role that the glymphatic system plays in the brain’s health and aging [124,126]. The aging impairs fundamental for brain physiology process of waste product removal. As a consequence, there is a progressing accumulation of toxic product and protein deposits in the senescent brain that impact all metabolic processes [87,120,124,125,126]. Importantly, the process of waste removal is particularly active during sleep [88]. Therefore, the age-related disorders of the circadian rhythm and sleep disorders are prognostic for most neurodegenerative diseases [77,88,125,126]. Accumulated intracellular and extracellular deposits worsen both functioning of individual neurons and neuronal networks. Kinetics of all vital neurochemical processes drop steadily, which escalates neurodegeneration. The brain’s and neuronal capability to repair molecular lesions also rapidly collapse. Neurons that have accumulated a large amount of damaged DNA and misfolded proteins, or those that no longer effectively repair DNA lesions enter the process of senescence and apoptosis [30,31]. Depending on which region of the brain is the most affected by aging and neurodegeneration, a characteristic set of clinical symptoms emerges.

There exists a physiological mechanism that combines in the brain local activity of neuronal networks with energy supply in health and pathology [127,128]. The functioning of the brain’s vascular system depends on the proper activity of neurons that, in turn, depends on effective blood flow. Both ischemia and abnormal blood vessel functioning are some of the basic causes of the progressive metabolic decline in the aging brain [116]. The neuronal dysfunctions closely correlate with the accumulation of blood vessel abnormalities, such as capillary basement thickening and endothelial hyperplasia, which contributes to a decrease in oxygen supply (hypoxia).

In the aging brain, the efficiency, and selectivity of the brain’s vascular bed and of the blood-brain barrier decline [123]. Micro damages to blood vessels and changes in the permeability of brain-protective barriers cause depletion or even blockage of the supply of substances necessary for the proper functioning of neurons. The inefficient or damaged blood-brain barrier causes undesirable substances, and pathogens can invade the brain tissue provoking local inflammation that intensifies degenerative and necrotic processes. Pathological effects of unsealed the blood-brain barrier and vascular microdamage are usually augmented by increased blood pressure and type 2 diabetes [129,130]. The latter pathology is the direct effect of neuronal deficit of ATP production, resulting in decreasing absorption of glucose and the general collapse of the brain’s energy metabolism.

## 5. Energy Metabolism and Neurodegenerative Disorders

The aging of the CNS is a complex process that seems to be predominantly triggered by the dysfunction of energy metabolism [1,19,21,29,31,32,67,68,92,100,101,130,131,132]. The effects of aging are particularly prominent in the nervous structures and functions that are most sensitive to energy deficits. Especially, the unique anatomy and physiology of the nervous structures, such as the cerebral cortex, hippocampus, and the basal ganglia, make them particularly prone to aging and neurodegeneration [133,134,135]. Therefore, memory, cognition, and movement control are the very first processes affected by brain aging. One should keep in mind that the degradation of these brain networks depends, however, on their life-long shaping and current physiological status. The brain structures that have been less active/used in ontogeny are eliminated first. As a consequence, an individual set of prodromal and clinical symptoms of neurodegenerative disorders can vary significantly. Depending on morphological differences between neuronal cells and networks, there are several primary targets of the brain’s aging and related deficient energy metabolism [70,89,90,93,100,133,136]. Although the etiology of neurodegenerative diseases seems still enigmatic, there is consensus for the impact of the decreased energy metabolism, excitotoxicity, and oxidative damage on their development [18,19,31,65,130]. Mitochondria, as energetic cellular centers, are particularly susceptible to energy deficit and oxidative stress. There is an increasing body of evidence that age-dependent damage and deterioration of mitochondrial respiratory enzymes are mainly responsible for brain aging [19,29,127,132].

Neurodegenerative diseases are disorders characterized by irreversible and progressive destruction of the structure and function of the brain [67,90,100,120,134]. This process usually begins in specific areas of the brain, and depending on it, cognitive deficits (Alzheimer’s disease, frontotemporal dementia) [130] or motor symptoms (Parkinson’s disease and Huntington’s disease) dominate in the clinical phase [79,93,94,136,137]. Usually, the occurrence of neurological symptoms is preceded by increasing metabolic dysfunctions, such as increased blood sugar and weight gain or loss [71,73], which is accompanied by changes in eating habits and preferences.

### 5.1. Proteinopathies and Alzheimer’s Disease

From a neuropathological perspective, Alzheimer’s disease is identified by the presence of neurofibrillary tangles in the brain, composed of intraneuronal fibrous aggregates of hyper- and incorrectly phosphorylated tau proteins, and extracellular accumulation of beta-amyloid [62,83,93,94,101,130,134]. Under physiological conditions, beta-amyloid is continually produced in neurons by the sequential action of two proteases: Beta and gamma secretases, which cleave the amyloid precursor protein (APP). This protein is synthesized in the endoplasmic reticulum and then transported to the plasma membrane. There, enzymes called secretases cut APP into bioactive fragments. Some of the cleaved APP fragments are transferred then to the vicinity of synaptic areas, where during the bioelectrical activity, follicular fusion occurs necessary for the release of neurotransmitters. Thus, APP appears to modulate interactions with intracellular signaling systems responsible for the growth of axons and dendrites and support for various functions involved in maintaining synapses. In adult brains, APP and its fragments function as sensing molecules. In response to the neuronal activity, they control cholesterol homeostasis, the supply of neurotransmitter carriers, and synaptogenesis. These processes are particularly important in large neurons, in which APP can act as a long-range sensor that transmits feedback information on synapse functioning and their activity back to the cell body.

In adult brain neurogenesis, APP affects neuroblast migration [138,139]. APP activity is intensified during the maturation of the brain and synaptogenesis in the processes of learning and memory. These observations suggest that APP plays a fundamental role in forming synaptic connections and shaping and maintaining neuromuscular junctions. Since protein synthesis precursor amyloid is regulated by synaptic activity, APP and fragments thereof can regulate neuronal lipid metabolism, necessary for regenerating the cell membrane and mitochondrial membranes, which structure is permanently exposed to microdamage (micropores) during the bioelectric activity of neurons [60].

The amount of beta-amyloid remaining in the brain tissue depends on both the level of neuronal activity and the efficiency of the brain’s waste removal. Both processes are energy-dependent. The decline in the efficiency of beta-amyloid removal leads to the accumulation of toxic oligomers and the formation of deposits damaging the structure and functions of the brain. The process of creating beta-amyloid deposits and neurofibrillary tangles commonly exists in every aging brain. The problem is the excessive accumulation of waste products in the aging brain. The aforementioned glymphatic system is the brain’s metabolite clearance system connected to the peripheral lymphatic system. Under physiological conditions, cerebrospinal fluid is pumped into the brain tissue in the rhythm of heart contractions, and then it returns to the ventricular system, while simultaneously flushing out wastes, including pathogenic beta-amyloid and tau proteins [23,87,126]. The glymphatic system is particularly effective during sleep when the clearance of harmful metabolites, such as amyloid β (Aβ) increases two-fold relative to the waking state [126]. That is why all sleep disorders are so harmful to brain physiology. Discovering novel strategies for optimizing and maintaining efficient brain waste clearance across the lifespan may, in the future, prove to be important for preventing cognitive decline and sustaining healthy aging [87].

The accumulation of toxic proteins exerts the most destructive effects on areas of the brain with the highest energy demands affected by progressing impairment of energy metabolism [134]. As a result of proteinopathy, hypoactive neuronal centers emerge that are destroyed then due to impaired metabolism [130]. Structures with high physiological activity, such as the cerebral cortex, hypothalamus, and striatum, are particularly prone to proteinopathy. Their functioning depends on several energy-consuming processes, such as maintenance of neuronal networks, neurogenesis, and waste removal. Disorders of these processes result from age-related hypothalamic dysfunction [135]. Pathologies include, in particular, the lateral periventricular nucleus of the hypothalamus, suprachiasmatic nuclei, tuber-mamillary bodies, and supraoptic nuclei, all that are responsible for the systemic control of energy metabolism [35,69,74]. This, in turn, strikes memory networks, such as the cerebral cortex, whichconsumes 80% of the brain energy [4,7,99].

### 5.2. Neurodegeneration in Parkinson’s Disease

The basal ganglia and the nigrostriatal system are the second area of the brain with high susceptibility for degenerative changes [71,97,131,138]. Many morphological factors contribute to this increased vulnerability. First, a relatively high energy supply must maintain an extremely extensive nigrostriatal movement memory network [71,131]. Motor learning and adaptive plasticity of the nigrostriatal network rely on the continuous exchange of a fraction of the striatal GABA interneurons [131,138,140]. Additionally, the high resting activity of the entire nigrostriatal system poses a great metabolic challenge. A relatively high energy cost is necessary to maintain such a network making it prone to neurodegeneration.

**Figure 2:**
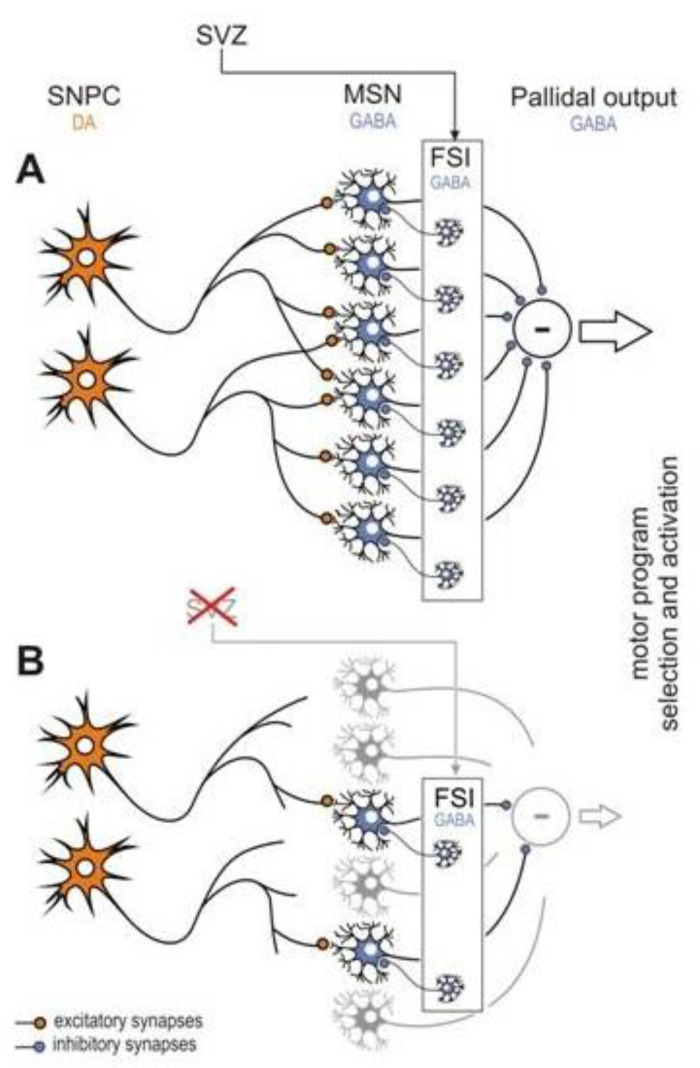
Model of the nigrostriatal interaction showing the physiology (**A**) and pathophysiology (**B**) of the striatum that may cause Parkinson’s disease [100]. In physiological conditions, the interaction is determined by the activity-dependent continuous turnover of GABAergic interneurons of the striatum. The fast-spiking interneurons (FSI) are particularly susceptible to apoptosis, thus, the physiological function of the striatum requires the continuous exchange of these interneurons. Normal activity of the striatum that is characterized by an elevated level of neurotransmitters (mostly GABA) and the glia-derived neurotrophic factor (GDNF) intensifies proliferation and migration of stem cells to the striatum. Both GABA and GDNF are chemoattractants for progenitor cells. The subventricular zone (SVZ) is a specialized brain area containing self-renewing GFAP^+^ astrocytes functioning as neural stem cells that generate new interneurons in both the striatum and olfactory bulbs throughout life. Age-related decline inneurogenesis is followed by a decline in the nigrostriatal interaction resulting in progressive withdrawal and eventually, disconnection of the dopaminergic input from the *substantia nigra pars compacta* (SNpc). This initiates a ’vicious circle’ cascade of pathological events resulting in a devastating decline of nigrostriatal interaction that leads to fatal damage of the FSI turnover and neurodegeneration of the DOPA neurons of the SNpc. In this pathological state, the striatum loses its control over the pallidal output, and clinical symptoms of Parkinson’s disease such as hypokinesia and rigidity are observed. The model reproduced with permission from Błaszczyk 2017 [131]. Copyright 2017 Acta Neurobiologia Experimentalis.

In neurons, most energy isspent on axonal transmission [4,34]. Such morphological factors as axonal fiber length, its myelinization, and axonal arborization are the main determinants of energetic neuronal demands.Single dopaminergic neurons of the *substantia nigra* can form up to thousands of synapses with GABAergic neurons of the striatum [131]. Moreover, the structure of nigrostriatal connections is dynamic and changes depending on individual motor activity, habits, and motor learning. Hipokinesia reduces energy supply for the nigrostriatal system that initiates the adaptive process of pruning of unused, and thus, unnecessary synapses [39,86]. The process of synaptic pruning is followed by axonal degeneration, and eventually, dopaminergic cell death [138,141]. Moreover, the entire GABA-ergic striatum is impacted by deficient dopaminergic input (Figure 2). Their decreased activity results in the decline of striatal neurogenesis [86,100,101,137,138,139,140] and reduced synaptogenesis both in nigrostriatal and striatal systems.

The main symptom of the nigrostriatal network reduction is increased muscle stiffness, and consequently, further reduction of motor activity in older adults [131,136]. Impoverished motor activity results in the reduced expression of glial cell-derived neurotrophic factor (GDNF) in the *striatum*, which is important for the synaptogenesis and the subventricular zone (SVZ) neurogensis, thus, functioning of the entire nigrostriatal network [68,71,131]. This causes a further reduction of dopaminergic synapses and the death of dopaminergic neurons of the *substantia nigra*. Increasing the nigrostriatal interaction inhibits neurogenesis of GABA interneurons in the subventricular zone that escalates the dysfunction of the striatum and the death rate of dopaminergic neurons of the substantia nigra [131,138,139]. Only fractional, compensatory nigrostriatal synaptogenesis delays the appearance of the first clinical motor symptoms of Parkinson’s disease until the majority (60–70%) of dopaminergic neurons are destroyed [131]. The results gathered up to date on the pathogenesis of idiopathic Parkinson’s disease suggest that the age-related decrease in the nigrostriatal interaction is the main cause of motor pathology during the disease. Therefore, the search for new therapies in Parkinson’s disease should now focus on slowing degenerative processes in the GABAergic striatum and restoring fully functional GDNF synthesis—the main chemoattractant for dopaminergic synaptogenesis and neurogenesis and migration of GABA interneurons [138].

Neurons of the brain’s functional networks are single-life cells. Only a marginal number of interneurons are continuously exchanged by the progenitor cells in the process of neurogenesis [71,138,139,140]. Neurogenesis, and the subsequent formation or modification of functional networks in the aging brain, are substantially impaired, due to progressing energy deficit and damages to the glymphatic system [137]. Understanding the process of neurogenesis in the secondary proliferative subventricular zone (SVZ) and identification of factors that impair the continuous renewal of the interneuronal network of the striatum and olfactory bulbs, should facilitate the development of new therapeutic strategies in neurodegenerative disorder [71,138,139,140]. The statement that interneurons are exchanged continuously in the adult brain raises the question of whether this process can be used therapeutically for treating Parkinson’s disease [71,100].

## 6. Perspectives for Treating Neurodegenerative Diseases

When analyzing the mechanisms underlying brain aging, it is hard not to notice that the cause of neurodegenerative diseases is the excessive death of nerve cells in specific areas of the brain. Knowing that the processes of apoptosis and necrosis are irreversible, one can only try to stop or slow down these processes for therapeutic purposes. The function of the dead neurons can no longer be recovered, but by slowing down the neurodegeneration process, we can activate plastic compensation mechanisms in the aging brain that will stop the avalanche progress of neurodegeneration.

Neurotoxicity and loss of neuronal processes induced by amino acids (glutamate and aspartate) is a hallmark of several neurodegenerative diseases, such as multiple sclerosis, Alzheimer’s disease, amyotrophic lateral sclerosis, Parkinson’s disease, and Huntington’s disease [46,51,142]. Besides, the excessive toxic concentration of glutamate around neurons may occur in hypoglycemic states. The excitotoxic effect of glutamate increases intracellular calcium ion levels, which trigger a cascade of pathological reactions that culminate in the death of nerve cells [30,31,102,103,104]. Studies show that intracellular calcium signaling is crucial for synaptic plasticity—the cellular mechanism of learning and memory. Therefore, calcium channel modulators and calcium signaling control are currently of interest to researchers in their potential use as neuroprotective mechanisms.

Latrepirdine acts as an inhibitor of NMDA receptors and voltage-gated calcium channels [143]. Latrepirdine modifies the permeability of mitochondrial membranes and thereby regulates calcium ion activity in mitochondria. The high concentration of calcium ions around neurons compared to their low maintained at the nanomolar level, the concentration in the cytoplasm, causes not only a high osmotic potential but also the electrical potential of divalent calcium ions. Moreover, the diameter of the hydrated calcium ions is the same as sodium ions. The accumulation of these three factors causes the electric field to rapidly change during the conduction of functional impulses on the cell membrane of neurons causes focal and rapid changes in the electrical conductivity of the membrane called electroporation [60,144]. The results of in vitro tests confirmed that the process of pore formation and subsequent clogging takes up to tens of seconds. We cannot explain how the phenomenon of electroporation works and why it has such a large time constant. We do not know why pore closing is controlled by ATP levels. The activity of calcium ions is particularly observed during the pore-closing stage. Research suggests that electroporation may be associated with secondary calcium signaling necessary to provide increased somatic and segmental energy metabolism in neurons [60]. This allows us to conclude that latrepirdine inhibition of glutamate-induced calcium signals may be used to protect neurons from excitotoxicity-induced apoptosis; thus, it can be a useful element of anti-neurodegenerative therapy.

GDNF signaling through the RET receptor and GFRα1 is of fundamental importance for maintaining the functional structure of the striatum and reconstruction of dopaminergic nigrostriatal projection. In animal models of Parkinson’s disease, the injection of GDNF into the striatum recovers the nigrostriatal function by creating new synaptic connections. Importantly, GDNF acts as a chemoattractant for both the axonal endings of SNPC neurons and for the activation and migration of SVZ progenitor cells [71,131,138]. It also promotes functional and morphological differentiation of neuroblasts reaching the striatum. GDNF through GFRα1 signaling participates in the growth of axons and promotes the formation of synapses on striatal GABAergic medium spiny neurons.In particular, GDNF activity also contributes to the rapid differentiation and incorporation into the striatal network of GABAergic interneurons. Unfortunately, progress in the development of new pharmacological based on GDNF is slow, since this factor does uncross the blood-brain barrier. There is, however, the emerging another possibility. There is a growing body of evidence that vitamin D increases tyrosine hydroxylase expression levels, suggesting that it can modulate dopaminergic processes. Vitamin D is a powerful inducer of endogenous GDNF, which may support the survival of dopaminergic neurons [145]. Thus, adjunctive vitamin D therapy may prove to be useful for treating Parkinson’s disease. Supplementation of vitamin D also helps in mitigating the effects of insulin resistance in neurons.

Importantly, the most straightforward GDNF therapeutic effect can be achieved by increasing the level of a patient’s physical activity [146,147]. Animals studies have shown an activity-induced increase of GDNF expression in several brain structures, including the *striatum*. This result suggests that the therapeutic effect of the neuroprotective and neurodegenerative GDNF can be simply controlled by physical activity. Physical activity causes in the nigrostriatal system synaptogenesis to increase, while limits the apoptosis in the *substantia nigra pars compacta* neurons. GDNF signaling by the RET receptor tyrosine kinase impacts the integrity and function of the blood-brain barrier, and thus, plays a potential role in the survival of neurons of the central nervous system. Finally, GDNF plays an important role in the activity of the microglia, which suggests that it can offer protection against neurodegeneration by blocking the inflammatory processes in the brain [37,148,149].

Nowadays, the recovery of efficient systemic energy metabolism is the most rational target for maintaining organismal homeostasis, physiology, and life. This hypothesis initiated an intensive search for strategies targeting brain and neurons energy metabolism in attempts to find anti-neurodegeneration therapy. It has been recently documented that NAD supplementation can effectively restore energy metabolism on both the cellular and organismal level [14,38,39,40,41,42,43,44,54,55,56,57,58,59]. Experimental results indicate that supplementing the brain with NAD precursors should ease the age-related functional brain deficits by restoring the physiological level of energy metabolism. This, in turn, should allow neurons to recoverATP production and slow down the process of neuronal aging and neurodegeneration. The newest studies have confirmed the therapeutic potential of supplementing NAD intermediates, such as nicotinamideriboside, providing a proof of concept for developing the new effective intervention [40,47,131,150,151].

NAD has a critical role as the substrate of NAD-dependent enzymes, including sirtuins and poly-ADP-ribose polymerases (PARPs) [110,111,112,114,118]. Whereas PARPs facilitate repair and maintenance of genomic integrity, the activity of sirtuins regulates protein quality control pathways, in particular catabolism of the unfolded proteins. Unfortunately, both PARPs and the sirtuins as NAD-dependent enzymes compete with ATP for the same limited and decreasing with age the intracellular pool of NAD. Each limitation of the ATP synthesis decreases glycolysis, which is the entry pathway of cellular energy metabolism. Therefore, supplementation of NAD intermediates, especially those that cross the blood-brain barrier, can protect firstly from various age-related neurodegenerative disorders. Supplementation of these intermediates appears to restore NAD levels in both the nuclear and mitochondrial compartments of neurons [40,58]. The results of the research to date seem to confirm that NAD-targeted therapy is effective, but only in the early stage of neurodegenerative processes [131]. Additionally, it must be combined with physiotherapy that activates the involved brain area(s) [146,147,152]. In summary, two hundred years of intensive research and efforts concluded finally in a novel approach to brain aging and neurodegeneration that give great hope for millions of present and future patients with neurodegenerative diseases.
